# P-125. A Single-Center Descriptive Analysis of Cryptosporidiosis in Immunocompromised Patients

**DOI:** 10.1093/ofid/ofae631.330

**Published:** 2025-01-29

**Authors:** Jishna Shrestha, Inderjit Mann, Pablo C Okhuysen

**Affiliations:** MD Anderson Cancer Center/ Baylor College of Medicine, Houston, Texas; Lehigh Valley Health Network, Allentown, Pennsylvania; The University of Texas MD Anderson Cancer Center, Houston, TX

## Abstract

**Background:**

Cryptosporidiosis is usually a self-limiting infection caused by a protozoan, *Cryptosporidium spp*. In immunocompromised, especially with T cell dysfunction, it can cause severe, potentially life-threatening infection. There is limited literature on cryptosporidiosis in patients with malignancies. We aim to look for differences in patient characteristics amongst acute (< 2 weeks) and chronic (≥ 2 weeks) cryptosporidiosis in patients with cancer.

**Table 1:**
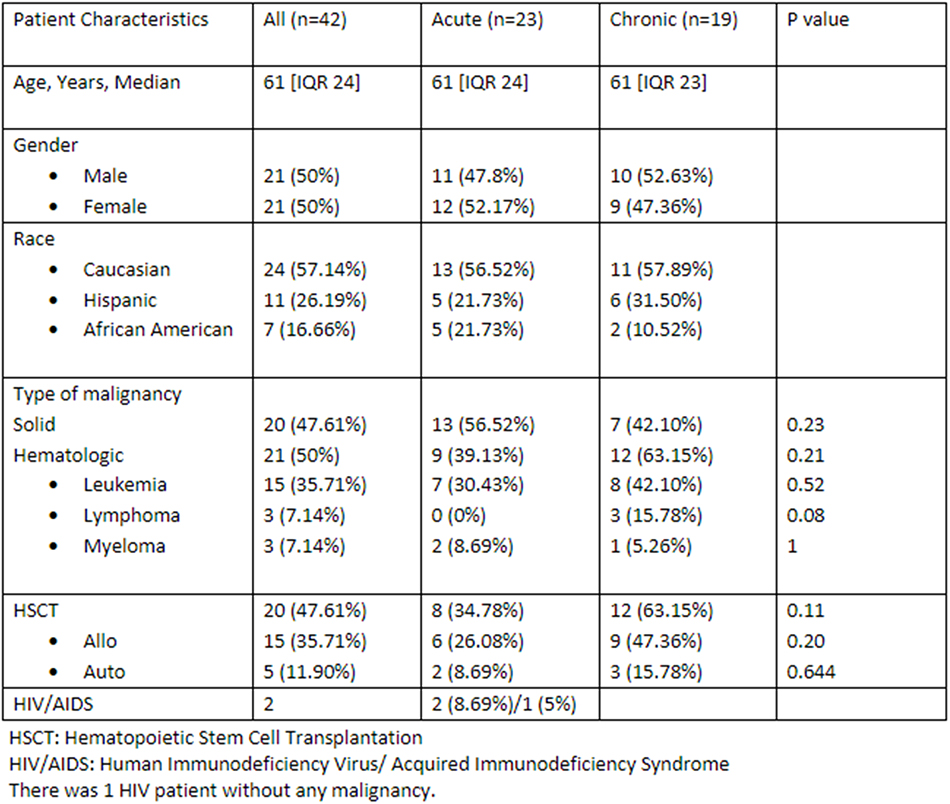
Baseline Characteristics of Study Population

**Methods:**

A single-center, retrospective chart review on patients diagnosed with Cryptosporidiosis with the help of Gastrointestinal Multiplex (Biofire) was conducted at UT MD Anderson Cancer Center between November 2016 and November 2023. Data was collected from EPIC electronic medical records and analyzed using Fisher’s exact test.

**Table 2:**
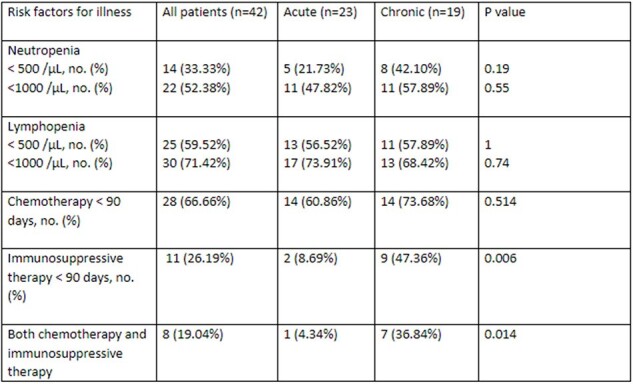
Risk factors for Illness

**Results:**

We identified 55 cases of cryptosporidiosis over 7 years. The date of first diagnosis was included in cases with multiple episodes. Among the 55 patients, 13 were excluded due to later confirmation of false positive results. Of 42 patients, the median age was 61 [IQR 24], 20 (48%) had non-hematologic malignancy and 21 (50%) had hematological malignancy [8 (19%) AML, 4 (9%) ALL, 3 (7%) MDS, 3 (7%) Lymphoma, 3 (7%) Myeloma]. 48% had hematopoietic stem cell transplantation, among which 35% were acute and 63% chronic. Within the last 90 days, 61% and 9% of acute cases, and 74% and 47% of chronic cases had received chemotherapy (p value= 0.514) and immunosuppressants (p value= 0.006), respectively. The median duration of symptoms prior to diagnosis was 3 days. Severe neutropenia was observed in 22% of acute cases and 42% of chronic cases, while severe lymphopenia was present in 57% of acute and 58% of chronic cases. Approximately 17% exhibited co-infection with other enteric pathogens, with most infections occurring in March, followed by October.

**Table 3:**
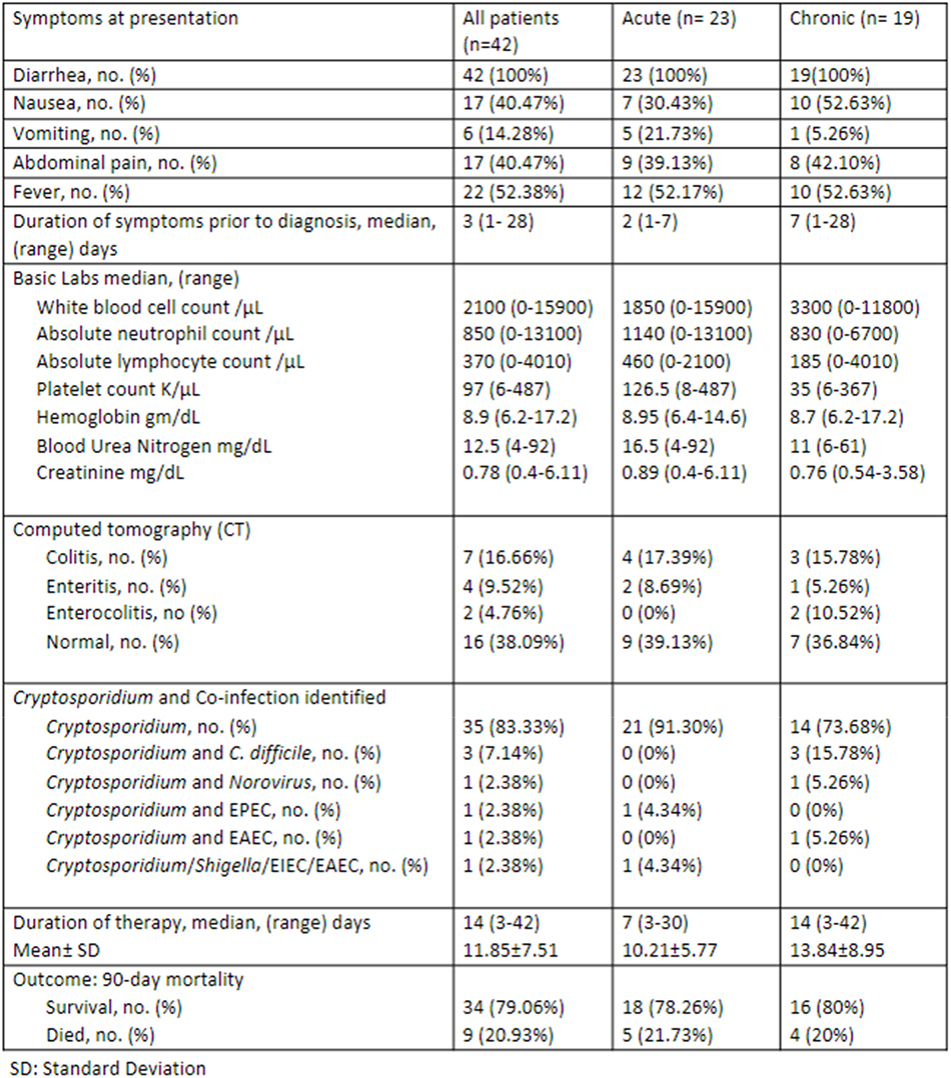
Clinical Presentation with laboratory and imaging, and Duration of therapy

**Conclusion:**

In patients with cancer and cryptosporidiosis, co-infection with a second enteropathogen was common, and no seasonality was observed. The use of immunosuppressants was associated with diarrhea lasting more than 2 weeks.

Cryptosporidiosis cases based on month

**Figure:**
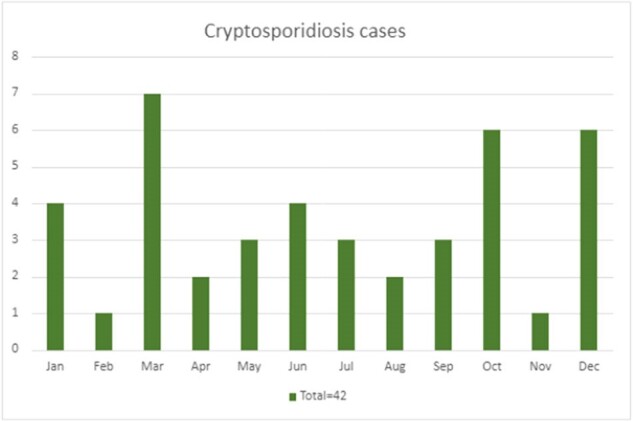

**Disclosures:**

**Pablo C. Okhuysen, MD**, Astra Zeneca: Stocks/Bonds (Public Company)|Beam therapeutics: Stocks/Bonds (Public Company)|Biontech: Stocks/Bonds (Public Company)|Deinove Pharmaceuticals: Grant/Research Support|Ferring Pharmaceuticals: Honoraria|Glaxo Smith Kline: Stocks/Bonds (Public Company)|Haleon: Stocks/Bonds (Public Company)|Johnson and Johnson: Stocks/Bonds (Public Company)|Melinta pharmaceuticals: Grant/Research Support|Moderna: Stocks/Bonds (Public Company)|Napo Pharmaceuticals: Advisor/Consultant|Napo Pharmaceuticals: Grant/Research Support|Napo Pharmaceuticals: Honoraria|Novavax: Stocks/Bonds (Public Company)|Pfizer: Stocks/Bonds (Public Company)|Summit Pharmaceutical: Grant/Research Support

